# DNA deformability changes of single base pair mutants within CDE binding sites in *S. Cerevisiae *centromere DNA correlate with measured chromosomal loss rates and CDE binding site symmetries

**DOI:** 10.1186/1471-2199-7-12

**Published:** 2006-03-16

**Authors:** Brad Hennemuth, Kenneth A Marx

**Affiliations:** 1 Center for Intelligent Biomaterials, Department of Chemistry, University of Massachusetts Lowell, Lowell, MA 01854, USA

## Abstract

**Background:**

The centromeres in yeast (*S. cerevisiae) *are organized by short DNA sequences (125 bp) on each chromosome consisting of 2 conserved elements: CDEI and CDEIII spaced by a CDEII region. CDEI and CDEIII are critical sequence specific protein binding sites necessary for correct centromere formation and following assembly with proteins, are positioned near each other on a specialized nucleosome. Hegemann *et al. BioEssays *1993, 15: 451–460 reported single base DNA mutants within the critical CDEI and CDEIII binding sites on the centromere of chromosome 6 and quantitated centromere loss of function, which they measured as loss rates for the different chromosome 6 mutants during cell division. Olson *et al*. *Proc Natl Acad Sci USA *1998, 95: 11163–11168 reported the use of protein-DNA crystallography data to produce a DNA dinucleotide protein deformability energetic scale (PD-scale) that describes local DNA deformability by sequence specific binding proteins. We have used the PD-scale to investigate the DNA sequence dependence of the yeast chromosome 6 mutants' loss rate data. Each single base mutant changes 2 PD-scale values at that changed base position relative to the wild type. In this study, we have utilized these mutants to demonstrate a correlation between the change in DNA deformability of the CDEI and CDEIII core sites and the overall experimentally measured chromosome loss rates of the chromosome 6 mutants.

**Results:**

In the CDE I and CDEIII core binding regions an increase in the magnitude of change in deformability of chromosome 6 single base mutants with respect to the wild type correlates to an increase in the measured chromosome loss rate. These correlations were found to be significant relative to 10^5 ^Monte Carlo randomizations of the dinucleotide PD-scale applied to the same calculation. A net loss of deformability also tends to increase the loss rate. Binding site position specific, 4 data-point correlations were also created using the wild type sequence and the 3 associated alternate base mutants at each binding site position. These position specific slope magnitudes, or sensitivities, correlated with and reflected the underlying position symmetry of the DNA binding sequences.

**Conclusion:**

These results suggest the utility of correlating quantitative aspects of sequence specific protein-DNA complex single base mutants with changes in the easily calculated PD-deformability scale of the individual DNA sequence mutants. Using this PD approach, it may be possible in the future to understand the magnitude of biological or energetic functional effects of specific DNA sequence mutants within DNA-protein complexes in terms of their effect on DNA deformability.

## Background

**Figure 1:**
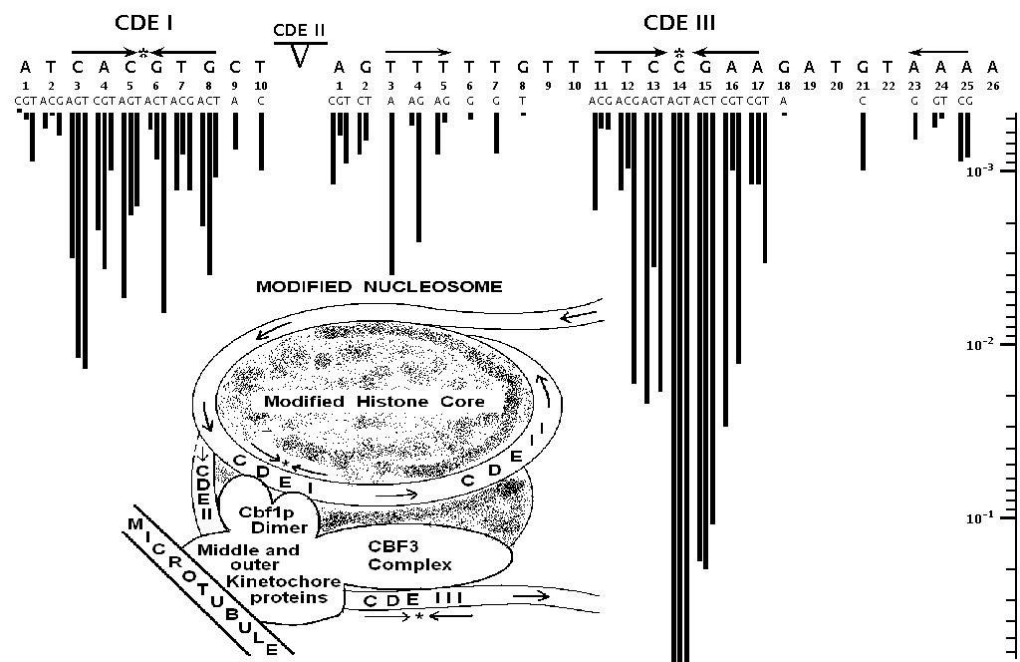
**CDEI & CDEIII sequences, their associated mutant's chromosome loss rates, and approximate locations of the DNA-protein components in a centromere nucleosome model**. At the top the three main conserved wild type DNA sequences, CDEI, CDEII, and CDEIII are represented. The core palindromes are marked by bold arrows and the symmetry centers by asterisks. The flanking palindrome areas of CDEIII are indicated by lighter arrows. Below each of the wild type sequences, the different mutant bases tested for each wild type base position are shown. Beneath each mutant, the bold vertical line indicates it's relative log_e _(loss) rate, of magnitude indicated by the log scale shown. These data are recreated from Hegemann et al [7] Figure 3. The inset depicts a model of the yeast centromere showing the modified nucleosome formed by wrapping the CDEI site into close proximity to CDEIII. Also shown are their associated protein complexes that along with additional binding proteins form the kinetochore, which bind the spindle microtubule [2].

The centromeres in the yeast,*S. cerevisiae*, are organized on short DNA sequences (125 bp) on each of the 16 chromosomes consisting of 3 elements, CDEI, CDEII, and CDEIII, as shown in Figure 1. CDEI is a conserved 8 bp element (positions 1–8 in Figure 1) with a 6 bp palindrome. CDEII is an approximately 78–86 bp A+T base rich, length conserved sequence located between the other two elements. CDEIII is a 26 bp conserved element with a 7 bp core palindrome-bold arrows in Figure 1. Following assembly with sequence specific binding proteins, CDEI and CDEIII are positioned near each other on a specialized nucleosome (inset in Figure 1) and are critical assembly sites for additional specific protein-protein interactions necessary for correct centromere formation [1,2]. The Cbf1p protein binds as a homodimer to CDEI, while CBF3 is a multi-protein complex that binds to CDEIII. There is also evidence supporting a subsequent interaction between Cbf1p and CBF3 [3,4], although this may be mediated through additional proteins [5]. These proteins form part of the inner kinetochore structure that, with additional protein kinetochore layers, is attached to the microtubule [6]. The microtubule is part of a spindle apparatus in the dividing yeast cell that is responsible for correct segregation of each chromosome into the daughter cells during cell division.

In a detailed genetic mapping study of the centromere of chromosome 6, Hegemann et al [7] produced 67 single base DNA mutants within the critical CDEI and CDEIII binding sites and measured chromosome fragment loss rates for all the chromosome 6 mutants during mitosis. While no specific error ranges were presented in this study, the researchers indicated that repeated data points were within 10% of each other [8]. All possible single base mutants were tested for the 8 bp CDEI element which has a center of symmetry between positions 5 and 6. Similarly, all possible single base mutants were tested for positions 11–17 of the CDEIII element, which forms the highly conserved core 7 bp palindrome with a center of symmetry at position 14. These areas are referred to as the 'core' protein binding sequences in our study due to their base conservation and elevated loss rates. In Figure 1 below we show each base position and present the magnitude of the measured chromosome loss rate for each base mutant as vertical lines, where the length of each line is proportional to the log_e _(chromosome loss rate).

The current state of knowledge in understanding DNA-protein interactions posits that DNA sequence specific recognition by its cognate protein may happen by a combination of both direct and indirect readout of a DNA sequence [9]. Direct readout would correspond to specific functional groups in the DNA major and minor grooves, including tightly bound waters of hydration, interacting with specific amino acid residue features of the 3-D structured protein. Indirect readout would correspond to features such as characteristic distortions of the DNA sequence that may not be entirely sequence specific. A type of indirect readout of DNA is that characteristic of the deformation brought about by sequence specific protein binding. Olson et al [10] have used crystallography data on nearly 100 protein-DNA complexes to produce a type of indirect readout scale, the dinucleotide protein deformability energetic scale (PD-scale), that describes the average DNA deformability by sequence specific binding proteins. The relative positions of adjacent planar base pairs are calculated in terms of the 6 dimensional parameters: twist, roll, tilt, shift, slide and rise. From these calculations, each of the 10 unique dinucleotides was assigned a single number that represents the multidimensional volume of energetic states found for that dinucleotide. That numerical PD-scale value for each dinucleotide is a representation of the average overall deformability for that dinucleotide by sequence specific binding proteins.

In this study, we have demonstrated novel correlations between the chromosome 6 single base pair mutants' chromosome loss rate data obtained by Hegemann et.al., [7], expressed in the log_e _form, and the PD-scale differences for each single base mutant calculated from the difference between each mutant and the wild type DNA. The correlations were observed for both CDEI and CDEIII binding sites. We also examined each binding site position's specific 4 data-point correlation, created using the wild type sequence and the 3 associated alternate base mutants' chromosome loss data for that position. Slope magnitudes from these position-specific correlations were found to be symmetric about the center of symmetry of both the CDEI and CDEIII sites. The slopes quantitatively represent symmetric energetic/functional aspects of the assembled centromere in vivo and reflect the underlying symmetry of the palindromic DNA binding sequences. Monte Carlo statistical controls were performed to demonstrate the significance of the observed correlations.

## Results and discussion

**Figure 2:**
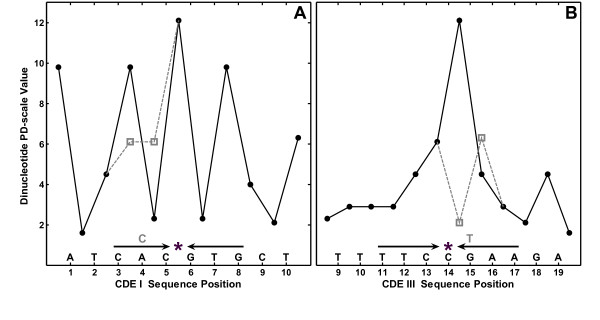
**PD-scale profiles of native CDEI & CDEIII compared with single mutant examples**. PD magnitude profiles of the positions in the core binding sites of wild type CDEI and CDEIII, shown as filled circles. The 2 PD values altered by a single selected base mutant in both core binding areas are shown as squares with the mutant base placed above the corresponding base in the wild type sequence.

Sequence specific protein-DNA binding events play many key roles in cells, ranging from dominant roles in the regulation of gene expression to critical roles in correctly segregating sister chromatids during cell division, the role relevant to the system we examine here. The ongoing efforts to leverage the growing numbers of high resolution X-ray crystallography and NMR structures to identify a useful DNA-protein 'interaction code' continues with modest success [11]. Some authors have described recognition in terms of direct and indirect readouts of DNA binding sequences by the protein. A type of indirect readout of any sequence is provided by scales that describe how the DNA is deformed by protein binding. One such scale, the simple PD-scale determined from these high resolution structure data, forms part of that effort to identify an 'interaction code'. Use of the PD-scale has achieved some successes; for example, in helping to understand the energetic signature of transposon target sequences [12] and receptor response elements [13]. Applying the PD-scale to the CDEI and CDEIII core binding sites and their single base mutants, we present in Figure 2 the PD-scale energetic signatures for each site. There is a clear significant difference between the two sites. CDEI has the characteristic seesaw deformability pattern observed in other protein binding sites [12] with a roughly uniform distribution of pyrimidine (Y) and purine (R) bases. It is also symmetric about the center of symmetry of the CDEI site. CDEIII however is more unusual with its poly-Y, poly-R half-site arms oriented toward a single critical peak at the center of the palindrome. The deformability peaks in both profiles correspond to the flexible YR dinucleotides that are frequently utilized in protein-DNA binding sites and are sometimes referred to as 'hinges.' [10] CDEIII DNA is the primary factor in determining centromere location [2,6]. However, this site possesses a relatively small number of conserved bases, leading some to speculate that additional indirect readout properties account for this specificity [14]. The isolated large deformability peak evident at the symmetry center of the palindrome is likely to play a key role in this recognition system.

### PD changes in mutant sequences correlate with measured chromosomal loss rates

**Figure 3:**
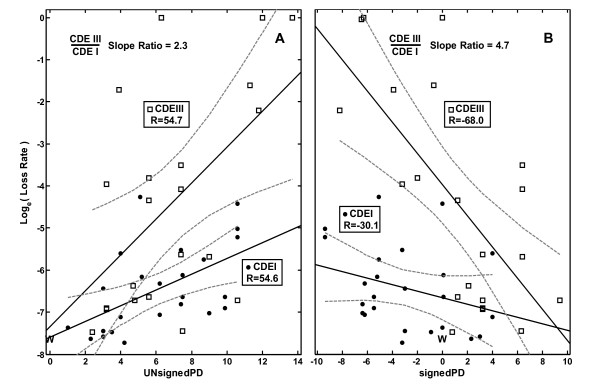
**Regression plots of chromosome loss rates vs PD changes for CDEI & CDEIII single mutants**. Scatter plots and regression lines for core binding sequences showing steeper slopes for CDEIII (open squares) as compared to CDEI (solid circles) for both (A) unsignedPD and (B) signedPD. Correlation values, R, are shown within text boxes. The position of the wild type data point is plotted as a 'W'. Dashed lines show 95% confidence intervals for average predicted values.

The signedPD and unsignedPD (see methods) are two simple but very different ways of quantitatively representing the change in DNA energetic deformability associated with protein binding to DNA due to a single base mutation in the binding sequence. The signedPD indicates the net change in deformability; the lower the number the greater the overall loss of deformability. By contrast, the unsignedPD indicates the total magnitude of change in deformability due to the mutation. The correlation results for both measure types are given in Table I. Associated regression plots for the most important core binding areas are shown in Figure 3. The unsignedPD results exhibited uniformly good, statistically significant, positive correlations in both core protein binding areas as well as for the entire mutant data group-labelled ALL. In general, the greater the magnitude of change of energetic deformability in either direction, the greater is the measured chromosomal loss rate. The overall correlation quality of the CDEIII core area is not as good as for CDEI and this is reflected in the regression P-values as well as visually in Figure 3A. The unsignedPD correlations tend to be evaluating two factors with respect to PD-scale perturbations: the relative loss rates at a specific position and the comparison of average positional loss rates. This latter factor could be thought of as the position specific sensitivity to mutation. Both factors are significant contributors to the overall correlations, but the extreme positional sensitivity variation of the data in CDEIII probably lowers the correlation quality to some degree.

It is worth noting that the intercepts of the regression lines from both these unsignedPD CDEI and CDEIII core areas yield values very close to the wild type chromosome 6 loss rate point (marked as 'W' in Figure 3A). The wild type point was not included in the determination of the correlations and slopes. We believe that the closeness of the intercepts to the true wild type chromosomal loss rate value, along with the magnitudes of the correlations, suggests that the PD-scale is an appropriate and valid method for representing mutant DNA sequence differences that bring about consequent energetic and functional changes for this DNA-protein system.

When we simply compare the raw signedPD data values associated with the two core area mutant groups, the differences are quite noticeable. The wild type average dinucleotide PD value for each site is about the same, but the average signedPD value for all mutants is about -3 for CDEI vs +1 for CDEIII. This is explained by the fact that CDEIII positions often have 2 of the 3 mutants introducing a YR hinge while many of the CDEI positions have 2 of 3 mutants eliminating YR hinges. Furthermore, the CDEIII position 14 and 15 mutants tend to eliminate the center YR hinge and therefore most have a high net loss of deformability. These mutants also have the highest loss rates (suggesting the biological importance of this YR hinge to CDEIII-protein binding and correct centromere function). Since just the opposite is true of most of the other CDEIII position mutants, this partly explains the exceptionally high signedPD correlation value in Table 1. This means that the poly-Y, poly-R CDEIII site creates a tendency for higher correlation values in the signedPD case as reflected in the Monte Carlo 0.059 probability of higher correlation than the actual value and leaves the general significance of the high signedPD correlation debatable. Indeed the other signedPD results presented in Table 1 showed much lower correlations. However, in contrast to the unsignedPD results, there is a clear tendency for these correlations to be negative. The only exception is the non-core group, which has the only positive slope and, as in the unsignedPD case, has regression values indicating that the correlation is not significant. Therefore, although expressed statistically somewhat weakly, these data suggest that using the PD-scale in the signedPD metric, exhibiting a net lowering of energetic deformability, correlates to a higher chromosomal loss rate.

The slopes of these regressions are a representation of the rate of change in log_e _(chromosome loss rate) per unit PD-scale change. In other words, they measure the binding site functional sensitivity to deformability changes averaged over the entire site for which mutants were generated and studied. The ratios of CDEIII to CDEI slopes, 2.3 for unsignedPD and 4.7 for signedPD, echo's the greater sensitivity of CDEIII compared to CDEI seen in the raw chromosome loss rates. Besides the raw loss rates of the Hegemann et.al., [1] study, many other experimental studies are in agreement with these slope ratios or binding site sensitivities, clearly indicating the greater importance of the CDEIII site compared to the CDEI site for correct centromere functioning [2,6,7]. This way of viewing the measured chromosomal loss rate data for different DNA sites provides an underlying structural/energetic basis for understanding them and calculating their magnitudes in terms of DNA deformation produced at those sites.

It is very clear from the group correlation values that the correlations are much stronger in the known conserved core protein binding centers as compared to strains with mutations in the less critical flanking areas, i.e. the non-core group. Although the mutant data is incomplete in these areas, the loss rates are varied and there are a few significant loss values. The lack of correlation here, in contrast to the core binding areas, may suggest a less critical role played by energetic deformability changes in these non-core DNA regions contributing to accurate biological function.

Dual variable regression models, using both the signedPD and unsignedPD, also reflect the overall weakness of the signedPD correlations. Analysis of variance reveals that none of the signedPD variable parameters are significant as an additional parameter except for the CDEIII group. There, the signedPD parameter is actually more significant than the unsignedPD, but both are very statistically significant. This last correlation has an R-value of 82.4; a P-value of 0.0027 reflects the significance of the unsignedPD parameter added as a second parameter.

### Monte Carlo simulation distributions demonstrate significance of delta PD sum correlations

The Monte Carlo analysis (see methods) was done as both a statistical crosscheck of the reported regression significance as well as to reveal trends or bias inherent in the data set. Dramatic differences between regression P-values and Monte Carlo P-values are an indicator of bias and question the validity of linear regression model assumptions. The Monte Carlo P-values are shown for both correlation and slope in Table 1 and generally have the same relative trends as the regression P-values. However, as noted above we see the stark contrast in P-values in the CDEIII signed case: 0.0007 vs 0.059 indicating significant data set bias not reflected in the linear regression values. Notably, from a pure statistical viewpoint, the best fit is the combined core area (CDEI & CDEIII) group using the unsignedPD metric. The regression P-value of 0.0004 and associated Monte Carlo value of 0.003 suggest the applicability of this approach to known sequence specific binding areas.

**Figure 4:**
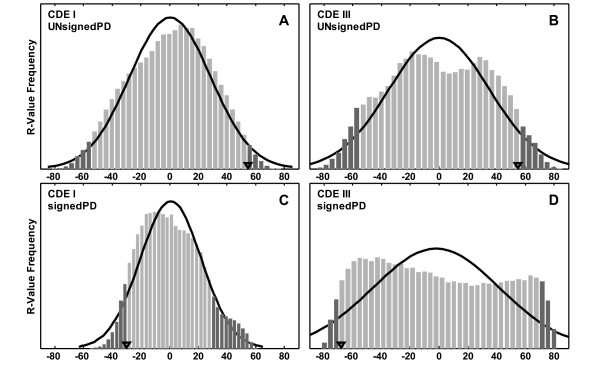
**Histograms of Monte Carlo R-value distributions for CDEI & CDEIII sites as a result of randomizing the PD-scale**. Histograms of the Monte Carlo distributions of R values are shown calculated from 10^5 ^randomizations of PD-scale values. In each histogram the actual value from the true PD-scale is marked by a triangle along the x-axis. Shaded dual tail areas correspond to probabilities of better correlations than the actual value reported in Table 1. The normal curve, superimposed on each of the histograms using the mean and standard deviation of the distribution, reveals the normal character of the CDEI distributions (A) & (C) and contrasts with the bimodal CDEIII distributions (B) & (D).

Histograms of the actual Monte Carlo distributions allow one to see the subtle bias reflected in the P-values. Figure 4 displays histograms of R-value distributions calculated for 10^5 ^randomizations of the PD-scale dinucleotide values for the core sites. The true R values using the correct PD-scale are marked on the x-axis for comparison. Note that the Monte Carlo values presented in Table 1 include both tail areas. For example, the A panel histogram area to the right of the triangle marker is only the positive part of the 0.016 probability area that contributes to a more significant correlation. The negative portion area is highlighted on the left side of the histogram.

The superimposed normal curve created from the mean and standard deviation of each dataset in panels A-D reveals that the CDEI distribution is closer to a normal form than the CDEIII and the unsignedPD distributions are closer to normal than the signedPD. Therefore, the least amount of bias attributed to the PD-scale is found in the unsignedPD CDEI case. It is striking that both CDEIII distributions show a tendency toward a bimodal form with the signedPD version having a more pronounced effect and a significant negative bias. The exact shapes of these distributions are a complex interplay between the mutant loss rate pattern and the overlaps of the PD-scale delta sum calculations.

**Figure 5:**
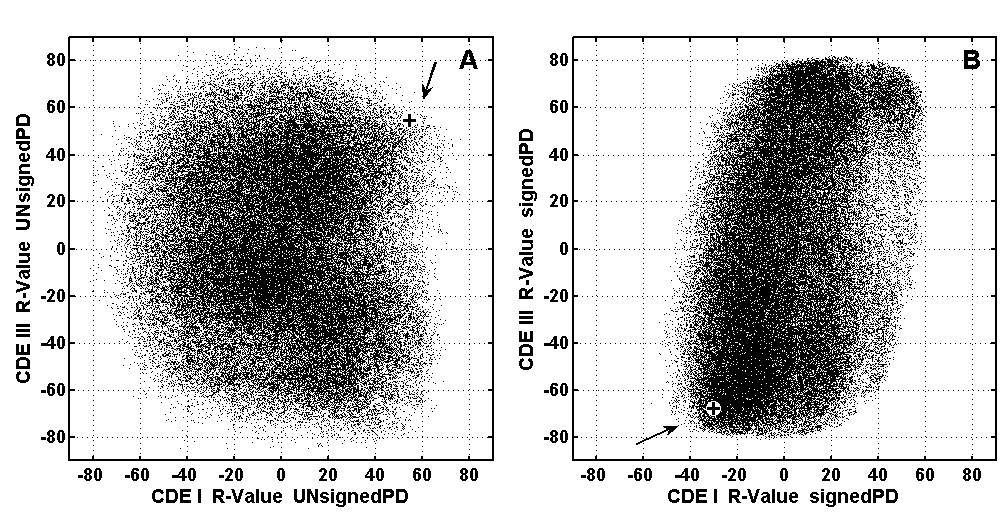
**Paired R-value distributions for CDEI & CDEIII from Monte Carlo results**. Monte Carlo distributions of paired R values from CDEIII vs CDEI core binding area regressions are presented for 10^5 ^randomized PD-scale values for unsignedPD (A) and signedPD (B). The actual values obtained from the true PD-scale are indicated by arrows and marked by '+' signs. The probabilities of better pair values in the respective quadrants are .0001 and .0044 for unsignedPD and signedPD respectively.

The CDEI and CDEIII paired scatter plots presented in Figure 5 also reveal the influence of the bimodal behavior of CDEIII. The unsignedPD distribution in Figure 5A is almost normal (circular), but reveals a subtle gap in the center right area. The signedPD distribution in Figure 5B shows a small but clear lean towards a diagonal orientation across the like signed quadrants. Although this latter behavior can be partly explained by both the bimodal CDEIII as well as the bias of both distributions toward the negative side, these plots are showing more information than is evident in the individual distributions. Namely, that the signedPD plot indicates a small bias toward like signed correlation values whereas the unsignedPD distribution does not. The real PD-scale R-values are marked in each scatter plot and can be seen to be extreme values relative to the distribution that the 10^5 ^Monte Carlo randomized PD-scale simulations produce. Expressed quantitatively, the probability of a Monte Carlo pair being more extreme than the real pair in their respective quadrants, that is having an equal or better R-value, is only 0.0001 and 0.0044 for unsignedPD and signedPD metrics, respectively.

### Double mutant groups

Three small double mutant groups were also tested [4,8] where a primary mutation is held constant and a secondary mutation is created. These groups can be evaluated as previously described by using the PD-scale delta sums corresponding to the secondary areas. The first group was the CDEIII position 15 base 'T' (15T) group and had as their second mutation a changed base in the CDEI position 3,4, or 5. The group consisted of 8 of the possible 9 CDEI mutant variants. The 15T group had no correlation using unsignedPD and a signedPD correlation value of 62.3 with a positive slope of 0.0262. The signedPD correlation was of poor quality; the regression P-value was 0.099. The second group was the CDEIII position 18 base 'A' (18A) group and consisted of the three possible CDEIII position 15 secondary mutations. Here we simply note that this group's 4 point unsigned position specific plot is a bit better than the single mutant version and maintains the general correlation trends previously discussed. The last group consists of 2 CDEIII double mutants which also follow previously discussed unsignedPD correlation trends. Although the first group results are contrary to the previous findings, it should be noted that this group is comprised of only 8 data points, and includes a high loss rate primary mutant. Use of the latter mutant forces an alternate loss rate assessment method to be used and involves mutations in both binding sites, which may introduce additional energetic factors in the chromosome loss rates involving changes in protein-protein interactions. Thus, these limited double mutant data do not provide a clear test of the utlility of the PD-scale representation.

### Position specific binding site sensitivities possess symmetric features mirroring the binding site sequence

**Figure 6:**
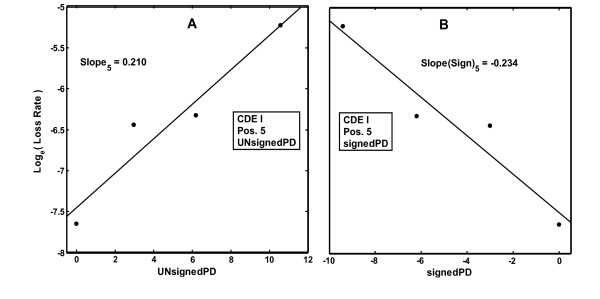
**Position specific regression plots for CDEI position 5**. Representative examples of two, position specific, 4 data-point linear correlations along with the regression slopes are presented. These are comprised of the 3 associated alternate base mutant data points along with the wild type data point for the CDEI position 5. This position possesses unusually good correlations for both unsignedPD (A) and signedPD (B) plots.

**Figure 7:**
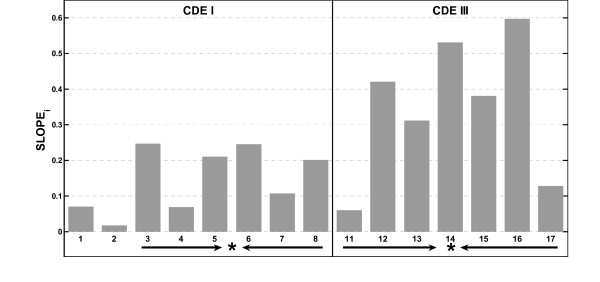
**Symmetric position specific regression line slope magnitudes for CDEI & CDEIII sites**. A bar chart of the position specific regression lineslopes from plots of log_e _(chrom. Loss rates) vs. unsignedPD change for both CDEI and CDEIII sites. Symmetry centers for both sites are marked with an asterisk and palindromic regions with bolded arrows. In the case of CDEI, symmetry is between positions 5 and 6. For CDEIII, symmetry centers on position 14, the central conserved cytosine.

Both CDEI and CDEIII DNAs contain palindromes. Consistent with these features are the following facts. CDEI is known to be a binding site for a homodimer protein; the loss rate magnitudes of CDEIII reflect the DNA symmetry; and both sites possess symmetric PD representations. Therefore, we examined the chromosome loss rates vs unsignedPD in a position specific way in both CDEI and CDEIII in order to see if the DNA deformability dependence we already observed also reflected this sequence symmetry. The slopes of the position specific correlations reflect the general sensitivity of that position to changes in energetic deformability. An example of these 4 point regressions are given in Figure 6 for CDEI position 5. Note that both the unsignedPD and signedPD correlations shown for position 5 are quite good. However, they are not representative of the entire group of positions as these regressions are quite varied in terms of significance. Nevertheless, as a group the unsignedPD 4 point correlations are uniformly more significant than the signedPD 4 point correlations. As is readily apparent in Figure 7 where the unsignedPD data is presented, the slope magnitudes for each site position are symmetric about the center of symmetry of each binding site. In the case of the CDEI palindrome, similar slope magnitudes exist for position pairs (5,6; 4,7; 3,8) that are symmetric about the center of symmetry of this sequence. For the CDEIII palindrome, the slope magnitudes are similar for position pairs (13,15; 12,16; 11,17), symmetric about position 14, the central conserved cytosine that is the exact center of symmetry. As we have already discussed in Figure 2, the cytosine at position 14 is the most highly deformable of all site positions in CDEIII. It is thought to be the position where critical protein interactions occur involving the p64 and p58 proteins [3,14]. That these slope magnitudes and position symmetry trends, calculated from PD-scale changes for position specific mutants, reflect their underlying DNA binding site symmetries provides strong support for the PD change approach being a useful method for understanding quantitative experimental aspects of DNA-protein interactions. Should sufficient confidence be gained in applying the PD approach to other systems, it may be possible in the future to make informed speculations about unknown aspects of a specific protein binding system based upon the behaviour of single base mutants in the underlying DNA binding site.

The overall position specific symmetry for both core sites can be described with one number by using the six pair relative closeness sum (see methods). The value can range from 0 to 6, the lower the number, the more symmetric the result. For the unsignedPD representation, this sum is 1.02, indicating a highly symmetric set of slope values for the combined core sites. Monte Carlo techniques using random PD-scale values as described in methods were used to count the number of values less than the real value, giving a probability of obtaining better overall symmetric patterns than the actual. It is understood that these unsignedPD 4 point regressions have a natural tendency to produce significant positive correlations and, even worse, the slope magnitudes would tend to reflect the combined loss rates of the 3 mutants. Yet despite this, the computed Monte Carlo probability for a more symmetric value is a relatively low 0.012 and this is conservative, as it allows for a fairly wide range of individual pair 'relative closeness' values. It drops to 0.007 if all six pair values are required to be no worse than the worst actual single pair value. Therefore, we see the single site position changes in log_e _(chromosomal loss rate) with respect to sensitivity to energetic deformability significantly reflecting the interactive protein-DNA symmetry of these systems.

The same symmetry analysis using the corresponding values for the signedPD yield much poorer results than for unsignedPD. The individual position specific slopes are less significant, and the overall symmetry is much worse; the six pair symmetry value is 2.59. Therefore, from a number of results we have presented, the unsignedPD is the more useful representation, producing significant correlations of the log_e _(chromosome loss rates) dependence on changes in the binding site deformability for both CDEI and CDEIII.

### Applicability to other systems

It is clear from the mutant loss rates in Figure 1 that the CEN6 wild type CDEI & CDEIII sequences possess superior levels of function compared to all the single base pair mutant sequences. In order to carry out their function, these native sequences must fulfill multiple energetic requirements that are dynamic in nature. The centromere formation process involves many proteins, some binding to both DNA as well as other proteins. Then, the resulting DNA-multiprotein complex must sustain the energetic forces of the chromosome separation process. Since the DNA dinucleotide based PD-scale represents the average deformability energetics expressed by dinucleotides involved in sequence specific protein binding, it is not that surprising to find general correlations between the magnitude of the energetic perturbations associated with the mutants and their resulting loss of function. While we have found that the PD sensitivity is roughly doubled in the CDEIII site, the unsigned PD regression combining all 45 mutants in these two core sequences with clearly different PD-scale signatures is still very significant. The low regression p-value implies the effects of PD change are relatively close in magnitude and highlights the general applicability of the PD-scale to this system. Our study (data not shown) using the Cbf1p/CDEI binding constants [15] shows correlation at selected DNA site positions. However, using this simple approach with all preserved site positions does not provide an overall significant level of correlation. Currently, we do not understand the reason for this. Certainly the binding constants for Cbf1p/CDEI complexes are quantities of far simpler physicochemical systems than are the DNA-multiprotein complexes involved in the *in vivo *chromosome segregation functional data we analyzed in this study. We do not yet know whether the types of correlations observed here will be a general type of behavior found in different DNA-protein systems where functional or energetic measurements of complexes possessing single base mutations have been carried out. We are interested in determining whether the PD change effects for other completely different DNA-protein systems exhibit correlations and also whether they are roughly linear as we observed here.

## Conclusion

The PD-scale represents DNA dinucleotide based deformability energetics by sequence specific protein binding. We have investigated the use of calculating changes in the PD value of single base mutant sequences relative to the wild type sequence to demonstrate correlations with measured chromosome loss rates for these single base mutants within the core CDEI and CDEIII protein binding sites in the centromere of yeast chromosome 6. We have produced novel results that lead us to the following conclusions. The greater the magnitude of change in energetic deformability of a given mutant, the greater is its measured chromosome loss rate. Generally this is linear for the rate of log_e _(chromosome loss rate) increase per PD-scale unit change, with the value for CDEIII being over twice that of CDEI. This higher deformation sensitivity reflects the conclusions from the data presented in Table 1 and agrees with numerous experimental studies, indicating that CDEIII is more critical to correct chromosome segregation than is CDEI. A net decrease in energetic deformability tends to correlate to a higher loss rate. This is strongly expressed in the CDEIII site. The position specific site sensitivities, or slope magnitudes, reflect the underlying sequence symmetries of these two sites. Taken together, these data suggest that the PD-scale representation of the deformability energetics of a DNA sequence, is an important simple attribute of the DNA sequence that could be used in future studies to quantitate and understand the functional consequence of alterations in a DNA recognition sequence upon interaction with its sequence specific recognition protein.

## Methods

We used the PD-scale of Olson et. al. [10] in the following way. The single base change in a mutant causes two adjacent dinucleotide PD-values to differ from the wild type (Figure 2). Let delta1 and delta2 be these differences, calculated by Mutant – Wild Type. Then delta1+delta2 is the signed PD-scale delta sum (signedPD), and is the net directional change in the PD scale attribute of the mutant with respect to the wild type. Similarly, |delta1|+|delta2| is the unsigned PD-scale delta sum (unsignedPD), and is the magnitude of the PD scale change. Each mutant's PD-scale delta sum is paired with the natural log of the chromosome loss rate forming a data point in our plots. The PD-scale delta sums and the log_e _(chromosomal loss rates) are used as the predictor and response values, respectively, for correlations using various groupings of these data points. Two types of groupings were used: larger groups of mutant data points without inclusion of the wild type data point and position specific, 4 data-point groups, comprised of the 3 associated alternate base mutant data points for a single position along with the wild type data point.

All of the correlations used standard linear techniques with a constant term. For each group, two separate regressions were performed: one using unsignedPD and one using signedPD. Additionally, 2 variable linear regressions were done for the larger groups using both signedPD and unsignedPD as predictor variables. While P-values for all parameters in the single and dual variable regressions were produced, control Monte Carlo based P-values were also calculated. This Monte Carlo method first randomly reassigned the 10 PD-scale values to the 10 unique dinucleotides and then performed the same correlations done with the real PD-scale values. This randomization process was repeated 100,000 times producing a distribution of R-values and, independently, slope values for each specific real-value regression performed. From these distributions, the probabilities of encountering a better correlation or a steeper slope than the actual values were obtained.

The position specific data are mainly only applicable to the core palindromic areas in CDEI & CDEIII, where all 3 mutants at the following positions were tested: CDEI 1–8 and CDEIII 11–17. The patterns of these regression values, relative to the center of symmetry, were the focus of our interest. Numerically, this symmetry is expressed by finding pairs of slope values close in magnitude. For CDEI, the symmetric position pairs are (5,6), (4,7), and (3,8); for CDEIII they are (13,15), (12,16), and (11,17). We expressed the relative closeness of two values, v1 and v2 as |v1-v2|/(|v1|+|v2|) allowing for a measure of the symmetry pattern by summing these 'relative closeness' values for all six pairs of symmetric positions. The value can range from 0 to 6, the lower the number, the more symmetric the result. Then, Monte Carlo techniques using random PD-scale values as described above were used to count the number of values less than the real value, giving a probability of obtaining better overall symmetric patterns than the actual.

The Mathworks' MATLAB^® ^version 7.0.1 programming software and statistics toolbox was used to calculate regressions and results.

## Abbreviations

Y – DNA pyrimidine base

R – DNA purine base

YR – DNA pyrimidine-purine dinucleotide

## Authors' contributions

BJH was primarily responsible for all aspects of the manuscript including all conceptual statistical work and writing. KAM was responsible for conceiving the project idea and was instrumental in its guidance, supervising the work presented, and writing and editing of the final manuscript.

## Figures and Tables

**Table 1:** Correlation and Monte Carlo data values

UNSIGNED PD					Monte Carlo	
Mutant Group	Data Points	R Value	Slope	Reg. P-Value	Prob.(Better R-Value)	Prob.(Steeper Slope)

CDE I Core	24	54.6	0.186	.0057	.016	.009
CDE III Core	21	54.7	0.427	.0103	.052	.044
CDE I & III	45	50.1	0.347	.0004	.003	.003
ALL	67	43.0	0.264	.0003	.020	.007
Non Core	22	11.3	0.018	.6100	.280	.460
						

SIGNED PD						

CDE I Core	24	-30.1	-0.08	.152	.098	.050
CDE III Core	21	-68.0	-0.37	.0007	.059	.011
CDE I & III	45	-22.9	-0.11	.130	.228	.282
ALL	67	-30.1	-0.12	.013	.083	.065
Non Core	22	15.4	0.02	.493	.206	.194

Results for plots of Log_e _(chromosome loss rate) vs UNSIGNED PD or SIGNED PD delta sums for various mutant groupings: CDEI Core = CDEI positions 1 thru 8; CDEIII Core = CDEIII positions 11 thru 17; Non Core = All other position mutants. The R-value is the signed correlation, the regression P-value is a statistical measure of the slope significance. The right 2 columns show the control P-value results from the Monte Carlo method (see text).
